# Supplementary material for: A multispecies corridor in a fragmented landscape: Evaluating effectiveness and identifying high-priority target areas
Source: PLoS One. 2023 Apr 13;18(4):e0283258. doi: 10.1371/journal.pone.0283258 (PMC10101518; doi:10.1371/journal.pone.0283258)
Supplement: S1 Appendix — For the 1034 felid samples and 51 bush dog samples, the location and zone (North-Central) are summed by species. For protected areas, the total area is reported in parentheses. (DOCX) [file pone.0283258.s001.docx]

**Appendix. Details of the 1,085 scat swabs with confirmed species identity.** For the 1,034 felid samples and 51 bush dog samples, the location and zone, north (N) or central (C), are summed by species. For protected areas, the total area is reported in parentheses.

| **Location** | **Zone** | **Jaguar** | **Puma** | **Ocelot** | **Southern tiger cat** | **Bush dog** |
| --- | --- | --- | --- | --- | --- | --- |
| Ejército Argentino (6,951 ha) | N | 2 | 2 | --- | 6 | 2 |
| Parque Nacional Iguazú (54,380 ha) | N | 24 | 13 | 27 | --- | --- |
| Parque Provincial (P.P.) Guardaparque H. Foerster (4,309 ha) | N | --- | --- | 6 | 6 | 1 |
| P.P. Puerto Península (6,900 ha) | N | 9 | 4 | 3 | 52 | 5 |
| P.P. Urugua-í (84,000 ha) | N | 13 | 9 | 17 | 37 | --- |
| Refugio Privado Aguaraí-mi (3,050 ha) | N | --- | 3 | --- | 22 | --- |
| Reserva de Vida Silvestre Urugua-í (3,243 ha) | N | --- | --- | 1 | 1 | --- |
| Reserva Nacional Iguazú (12,620 ha) | N | --- | --- | --- | 3 | --- |
| Reserva Natural Privada Yate-í (15 ha) | N | --- | --- | --- | 7 | --- |
| Reserva Privada Karadya (90 ha) | N | --- | --- | --- | 4 | --- |
| Reserva San Jorge (21,163 ha) | N | 5 | 3 | 5 | 22 | 1 |
| Reserva Yacutinga (539 ha) | N | --- | --- | 11 | --- | --- |
| P.P. Araucaria (92 ha) | C | --- | --- | --- | 1 | --- |
| P.P. Cruce Caballero (522 ha) | C | --- | 2 | 2 | 2 | --- |
| Reserva Privada Itaovy (80 ha) & Reserva Privada Yacutoro (200 ha) | C | --- | --- | --- | 3 | --- |
| Valle del Arroyo Alegría (8,000 ha) | C | --- | 2 | 2 | 39 | 1 |
| P.P. Cruce Caballero (522 ha) & Valle del Arroyo Alegría (8,000 ha) | C | --- | --- | --- | 2 | --- |
| P.P. Esmeralda (31,569 ha) & Reserva de Biósfera Yabotí (236,313 ha) | C | 3 | 19 | 28 | 56 | 5 |
| P.P. Piñalito (3,796 ha) | C | --- | --- | --- | 9 | 3 |
| Reserva Privada Yaguaroundí (400 ha) | C | --- | --- | --- | 5 | --- |
| outside protected areas | N | 17 | 14 | 37 | 197 | 20 |
| outside protected areas | C | 3 | 28 | 6 | 240 | 13 |
|  |  | **76** | **99** | **145** | **714** | **51** |
