# Supplementary material for: A multispecies corridor in a fragmented landscape: Evaluating effectiveness and identifying high-priority target areas
Source: PLoS One. 2023 Apr 13;18(4):e0283258. doi: 10.1371/journal.pone.0283258 (PMC10101518; doi:10.1371/journal.pone.0283258)
Supplement: Attachment — Submitted filename: PONE-D-22-23711_reviewer - AG comments.docx [file pone.0283258.s002.docx]

Manuscript	[Click here to access/download;Manuscript;DeMatteo et al (PLoS ONE 24-08-22).docx](https://www.editorialmanager.com/pone/download.aspx?id=31724591&guid=f248f153-8209-4090-91a1-a2ec2c801c8e&scheme=1)

1 A multispecies corridor in a fragmented landscape: evaluating the effectiveness and identifying high-
2 priority target areas

3

4	Multispecies corridor: evaluating effectiveness and priority areas

5

6 Karen E. DeMatteo^1,2,*,¶^, Orlando M. Escalante^3,4,&^, Daiana M Ibañez Alegre^3,&^, Miguel A. Rinas^5,¶^,
7 Delfina Sotorres^3,4,&^, and Carina F. Argüelles^3,4,¶^

8

9 ^1^ Washington University in St. Louis, Department of Biology & Environmental Studies, St. Louis,
10 Missouri, United States of America
11 ^2^ WildCare Institute at the Saint Louis Zoo, St. Louis, Missouri, United States of America
12 ^3^ Grupo de Investigación en Genética Aplicada (GIGA), IBS – Nodo Posadas, Universidad Nacional de
13 Misiones (UNaM) – CONICET, Posadas, Misiones, Argentina
14 ^4^ Facultad de Ciencias Exactas, Químicas y Naturales, Departamento de Genética, UNaM, Posadas,
15 Misiones, Argentina
16 ^5^ Ministerio de Ecología y Recursos Naturales Renovables, Posadas, Misiones, Argentina

17

18 * Corresponding author
19 E-mail: [karendematteo@outlook.com](mailto:karendematteo@outlook.com) (KED)

20

21 ¶ These authors contributed equally to this work.
22 & These authors contributed equally to this work.

23

24	**Abstract**

25 While Misiones, Argentina contains one of the largest remnants of Upper Paraná Atlantic Forest
26 ecoregion, one of the world’s biodiversity hotspots, only ~50% of this native forest is protected. Each
27 protected area is at risk of becoming an island of native forest surrounded by a matrix of altered
28 habitatstat due to ongoing land conversion. In an effort to maximize long-term connectivity between
29 existing protected areas, DeMatteo [1] used a multifaceted cost analysis to determine the optimal
30 location for the region’s first multispecies corridor using noninvasive data on jaguars (*Panthera onca*),
31 pumas (*Puma concolor*), ocelots (*Leopardus pardalis*), southern tiger cats (*Leopardus guttulus)*, and
32 bush dogs (*Speothos venaticus*). This work builds on this framework by integrating new field data that
33 broadens the scope of species-specific data across the region’s heterogeneous landscape, which varies
34 in vegetation, disturbance, human proximity, and protective status. In addition, two different land use
35 layers are compared across the distributions of the five carnivores, the overlap in their independent
36 distributions, and their relationship to the multispecies corridor. Interpretation of these land use data
37 to species-specific habitat suitability goes beyond DeMatteo [1], with a subdivision of suitability into
38 marginal and optimal areas. This refined scale allows a reanalysis of key areas in the multispecies
39 corridor, where connectivity was previously defined as at highly-at-risk, allowing for a more directed
40 development of management strategies. These analyses and their interpretation extend beyond
41 northern-central Misiones, as the threats are not unique to this region. The need to develop
42 management strategies that balance human-wildlife needs will continue to grow as humans expand
43 their footprint. The techniques applied in this analysis provide a way to identify key areas that require
44 specific management strategies, either through restoration, protection, or a combination of both.

45

# 46 Introduction

47 As the human footprint continues its expansion worldwide [2,3], there is a need to develop
48 management strategies that balance human-wildlife needs in the face of habitat fragmentation and
49 varied human activities across the landscape [4,5,6]. This includes understanding the effect of
50 expanding human demands on the landscape relative to the long-term survival of biodiversity, habitat
51 integrity, and ecosystem services [6,7,8,9,10]. One approach is the use of a Geographic Information
52 System (GIS) to conduct spatial analyses that identify wildlife corridors or areas that ensure
53 connectivity across fragmented landscapes, while minimizing the potential for human-wildlife conflict

54	[11,12].

55 Wildlife corridors are often designed using existing protected areas as a series of “stepping
56 stones” across the heterogeneous landscape [1,13,14,15,16,17], which requires understanding
57 whether these intermediary areas between protected areas are a barrier to species movement (high
58 ecological cost) or seen by the species as suitable, even if of lower preference (e.g., lower prey
59 abundance, less protected cover) [18,19,20]. Defining this suitability requires generating a surface of
60 resistance to animal movement, which depends on deciding which factors should be included and the
61 specific weights that should be assigned to reflect their effect (positive or negative) on animal
62 movement [21,22]. The method applied to develop the surface will vary depending on whether actual
63 movement data is available (e.g., resource selection function; 23,24,25,26] or not (e.g., species
64 occurrence, density estimates, expert opinions; 27,28,29]. The technique to model connectivity (e.g.,
65 circuit theory, network flow, path selection, least-cost past, least-cost corridor) will vary depending on
66 study scale and focal species [30,31,32,33,34,35]. This extends to defining the optimal or minimal
67 width for the corridor, with the need to balance its length and width [21,26,36].
68 Some corridors are designed with a focus on a single species, to protect coexisting
69 species through an umbrella effect, but this may fail to completely capture the varied ecological
70 requirements of coexisting species and ecological processes across the landscape [32,37,38]. Others
71 are based on multiple species, to capture the breadth of ecological requirements, but this
72 may generate results that underestimate connectivity for species highly restricted in their movements
73 [38,39,40]. A more comprehensive approach balances the trade-offs of these two methods and
74 accounts for variation across multiple species, with information on the most restricted species used to
75 weight final decisions [32,37]. DeMatteo [1] used this balanced method to define a multispecies
76 corridor in northern-central Misiones, Argentina that would almost double the protected area
77 (+400,000 ha) and secure an additional ~300,000 ha of native forest. Specifically, DeMatteo [1] defined
78 the corridor using the breadth of species-specific habitat preferences for five carnivores [jaguar
79 (*Panthera onca*), puma (*Puma concolor*), ocelot (*Leopardus pardalis*), southern tiger cat (*Leopardus*
80 *guttulus*), and bush dog (*Speothos venaticus*)], while ensuring the most restricted species (jaguar) was
81 effectively captured [18,41,42].
82 The native forest that the DeMatteo [1] multispecies corridor would protect is part of one of
83 the largest remaining tracts of the Upper Paraná Atlantic Forest ecoregion, one of the world’s biodiversity
84 hotspots [43,44], with Misiones containing almost 1.4 million ha [45]. Despite the small size of
85 Misiones, which occupies only 1% of the total area in Argentina, it is considered it’s National Capital of
86 Biodiversity (Ley Nacional 27494), a designation that recognizes it contains >40% of the country’s
87 biodiversity. However, only ~35%, or ~500,000 ha, of this unique forest is protected in a series of areas
88 that vary in size, adjacency, and degree of protection. The connectivity between protected areas and
89 long-term survival of its biodiversity is threatened by many factors, including ongoing habitat
90 conversion, forest degradation, an expanding network of roads, population growth in rural areas, and
91 poaching [1,46,47,48,49,50]. The native forest outside of protected areas exists in a matrix of
92 plantations, agriculture, and pastures, which continue to expand annually [1,20,45,51,52] (Figure 1). In
93 1999, the province of Misiones proactively developed a plan to create the Green Corridor [Corredor
94 Verde; Ley Provincial XVI N°60 (previously Ley Provincial N° 3631/99)], to protect this
95 unique ecoregion; however, over the last two decades, on-the-ground protection of the forest,
96 waterways and biodiversity has been varied and irregular, with the Green Corridor primarily existing
97 on paper.

98

### 99 Figure 1. Map of northern-central Misiones, Argentina with detection dog survey routes (2009 thru

100 **2018) shown relative to protected areas, major roads, towns, and the two land-use raster grids used**
101 **in spatial analyses:** a) land use was derived from a mosaic of Landsat-8 TM satellite images from 2015
102 [45] and b) land use was derived using the automated classification of satellite images from 2018 [53].

| 103 |  |
| --- | --- |
| 104 | The multispecies corridor modelled by DeMatteo [1] effectively provides a strategic starting point to |
| 105 | make the Green Corridor a reality. However, only ~40% of the 761 carnivore scats used to model this |
| 106 | corridor were located outside of protected areas or in those areas that are essential for connectivity |
| 107 | between existing “stepping stones” or protected areas. While this initial data represented a first for the |
| 108 | region in its sample collection across multiple species independent of habitat integrity, proximity to |
| 109 | humans, and degree of habitat protection, subsequent detection dog surveys in the northern-central |
| 110 | zone allowed the collection of an additional 324 scats from the five targeted carnivores, with almost |
| 111 | 80% of samples located outside of protected areas. This work will build on the framework established |
| 112 | by DeMatteo [1] integrating these new field data [26] for a comprehensive set of 1,085 scats located |
| 113 | across the corridor’s heterogeneous landscape (e.g., degraded forest, pastures, near human |

114 populations, large-scale agriculture, monoculture plantations) that can be used to ground-truth the
115 DeMatteo [1] multispecies corridor. This expanded analysis will not only allow confirmation that the
116 location of the corridor is optimal but also provides insight into the effect of sample distribution (e.g.,
117 in/out of a protected area) on the efficacy of corridor modelling. These analyses will expand beyond
118 DeMatteo [1], which looked only at suitable versus unsuitable habitat for the five carnivores, and
119 include optimal, marginal, and unsuitable habitat quantities. These finer divisions across the corridor
120 will be used to identify key regions in need of management strategies that promote human-wildlife
121 coexistence [49,54,55]. This last step is essential given that ~60% of the multispecies corridor is
122 privately owned, so balancing the need of humans to “live off” the land with the need for biodiversity
123 to live in the heterogeneous landscape is a must for the long-term conservation of the Green Corridor

124	[4,56,57,58,59].

125

# 126 Materials and Methods

## 127 Field Surveys

128 Data were collected during five surveys (2009, 2011, 2013, 2016, & 2018) conducted primarily during
129 the cool season (May-August) of Misiones, Argentina. The Ministerio de Ecología y Recursos Naturales
130 Renovables of Misiones (MEyRNR) issued all general permits related to our project in the province,
131 collection of samples in the multiple provincial parks, and export permits of samples for genetic
132 analyses. The Administración de Parques Nacionales of Argentina issued permits related to the collection
133 of samples in the national park.
134 Survey tracks consisted of two-lane paved roads, 1-2 lane dirt roads, established machete-cut
135 trails, illegal hunting trails, and animal trails. The team surveyed a total of 257 unique routes and
136 walked a total of 1,613.7 km [mean (SD)=6.28 (3.62) km; range=0.23-21.06 km per route; Figure 1]. The
137 total coverage in the northern zone (992.4 km, 61.5%) was slightly higher (371.1 km, 23%) than that of
138 the central zone (621.3 km, 38.5%). In addition to protected areas of native forest, surveys covered
139 unique habitats including private native forests, small-scale agriculture, monoculture plantations of pine
140 and eucalyptus, small communities with subsistence agriculture, pastures, and human-occupied areas.
141 Of the total distance walked, nearly half (747.1 km, 46.3%) was located outside of protected areas.
142 All carnivore surveys were completed using the same detection dog-handler team [1,20,51,60].
143 Detection dogs eliminate dependence on visitation rate to a specific location (e.g., camera trap) and
144 instead switches the focus to locating evidence (e.g., olfactory) associated with the species’ natural
145 behavior and movement patterns. Even in the rugged terrain of Misiones, Argentina, detection dogs
146 have been able to effectively search large geographic areas and locate samples from multiple species,
147 while ignoring samples from nontarget species that may be similar in their appearance or composition
148 [1,20,51,60]. The training of the detection dog, swabbing of scat for genetic analyses, collection of
149 scats and recording of field data were carried out using the same strategies made in all surveys

150	[1,20,51,60].

151

## 152 Genetic analyses

153 Scat swabs were processed using DNA extraction protocols and genetic analyses detailed in previous
154 studies [1,20,51]; however, a brief summary is provided here. DNA was extracted from two
155 independent swabs using a Qiagen (Venlo, Netherlands) DNeasy™ DNA extraction kit following a
156 modified protocol by Vynne [61]. To identify species, a 110-bp (171-bp with primers) carnivore-specific
157 region of mitochondrial cytochrome *b* gene (5′-AAACTGCAGCCCCTCAGAATGATATTTGTCCTCA-3′; 5′-
158 TATTCTTTATCTGCCTATACATRCACG-3′ [62) was amplified with a modified version of the protocols and
159 reagents of Farrell [62] and Miotto [63]. Amplifications were performed on a MyCycler Thermal Cycler
160 System (BioRad, Hercules, CA) in 25-µL volumes containing 2-µL DNA extract, 1× PCR Gold buffer
161 [Applied Biosystems, Foster City, CA] 0.3-µM forward and reverse primer, 200 µM each dNTP, 5-mM
162 MgCl2, 150-µg/mL BSA (Ambion^®^ - Life Technologies, Grand Island, NY) and 1-U Ampli*Taq* Gold DNA
163 polymerase (Applied Biosystems). The PCR profile consisted of 10-min denaturation at 95°C, followed
164 by 40 cycles at 95°C for 30 s, 49°C for 45 s, 72°C for 45 s, and a final 30-min extension at 72°C. Purified
165 PCR products were sequenced using the ABI PRISM BigDye Terminator v3.1 Cycle Sequencing Kits (ABI)
166 and analyzed in an ABI 3100 Genetic Analyzer (ABI). Sequences were edited and aligned using
167 Lasergene Seqman 8.1 (DNASTAR, Madison, WI) and compared with reference entries in GenBank
168 using the Basic Local Alignment Search Tool (BLAST; 64) to identify sequences from Neotropical species
169 that had high similarity and closely-matched sample sequences.
170 Of the 1,451 total samples collected, species identity was confirmed in 1,085 (74.8%) (S1
171 Appendix). In the remaining scat swabs, species identity was not possible due to low quantity/quality
172 DNA or urine contamination through scent marking animals. While over half of the samples were
173 identified as southern tiger cat (n=714, 65.8%), there were 145 (13.4%) ocelot, 76 (7.0%) jaguar, 99
174 (9.1%) puma, and 51 (4.7%) bush dog. While the proportion of samples found in the northern-central
175 zone were similar in puma (48.5% : 51.5%), southern tiger cat (50.0% : 50.0%), and bush dog (56.9% :
176 43.%), they were more skewed in jaguar (92.1% : 7.9%) and ocelot (73.8% : 26.2%), with the elevated
177 proportion in the northern zone likely associated with the higher levels of contiguous forest [20]. Exact
178 sample locations are not reported or displayed per government request as a precaution to protect
179 these threatened and endangered carnivores from targeted poaching.

## 181 Modeling the ecological niche

182 MaxEnt 3.3.3.k was used to model ecological niches for the five carnivores and evaluate habitat
183 suitability [65], as it has been reported to perform consistently better than other algorithms [66,67]
184 and is effective at working with small numbers of presence-only samples [67]. Models were fit using
185 auto features and default parameters for regularization multiplier (1), convergence threshold (1.0E-5),
186 and background points (10,000) [68,69]. For each species, models were tested by randomly
187 withholding 25% of presence localities and running 15 subsample replicates with the average output
188 used for interpretation. We generated a logistic output, which gives the probability of species presence
189 on a scale of 0 to 1 and has been shown to improve model performance via model calibration of output
190 values and corresponding suitability [68].
191 Applying threshold or “cutoff” values to the logistic output of MaxEnt allows it to be converted
192 to predictions that allow habitat suitability to be evaluated. First, each model was converted to a
193 binary prediction where habitat was defined as suitable (high probability of species’ presence) or
194 unsuitable. Ecologically, values equal to or greater than the defined threshold value can be interpreted to
195 contain cells that are predicted to be at least as suitable as those where the species was identified
196 present. We compared the extrinsic omission rate and proportional predicted area (a proxy for
197 commission rate) at several logistic thresholds [e.g., minimum training presence (MTP), fixed
198 cumulative value 1 (FCV1)] [65,70,71], with our criteria to have an omission rate of zero but set lower
199 restrictions on the size of the potential predicted area. Second, the suitable habitat for each species
200 was subdivided into marginal and optimal habitats using the maximum training sensitivity plus
201 specificity (MTSS), a threshold noted to be most appropriate at defining optimal habitats in presence-
202 only models [70,72,73]. Ecologically, marginal habitat corresponds to values equal to or greater than the
203 binary threshold but less than the MTSS threshold; whereas, optimal habitat corresponds to values
204 greater than the MTSS threshold.

## 206 Predictor variables for the ecological niche model (ENM)

207 While DeMatteo [1] used a single land-use layer from 2009 [74], this work compares two data sets that
208 have a stronger fit to the later years of data collection (2009 – 2018), which effectively captures the
209 additional forest loss and land conversion across the landscape (Figure. 1). Model 1 used a land use
210 derived from a mosaic of Landsat-8 TM satellite images from 2015 [45]. Model 2 used a land use
211 derived from the automated classification of satellite images from 2018 [53].
212 Using ArcMap 10.8, a neighborhood analysis with land use type used focal statistics, at a 30 m ×
213 30 m resolution, to characterize conditions in neighborhood cells (Table 1) independently for each land
214 use layer. The landscape heterogeneity index is based on the Shannon-Wiener diversity index of
215 habitat type diversity, where higher heterogeneity values represent strongly anthropogenic landscapes
216 (i.e., a great number of human modified landscapes in a small area) [75]. A neighborhood scale of 333
217 cells x 333 cells was used to represent a home range of ~100 km^2^ (99.8 km^2^) for the jaguar
218 [76,77,78,79], puma [79,80], and bush dog [81]. A neighborhood scale of 150 cells x 150 cells were used
219 to represent a home range of ~20 km^2^ (20.25 km^2^) for the ocelot [76,79,82,83] and southern tiger cat
220 [83]. While both of these values are more conservative than DeMatteo [1], which used home ranges of
221 100 km^2^ and 150 km^2^ for all five carnivores, they are believed to provide a more accurate measure
222 given the scale of these analyses.

### 224 Table 1. Summary of predictor variables tested and used in the two species-specific ecological niche

225 **models generated in this work.** Model 1 used land use derived from a mosaic of Landsat-8 TM satellite
226 images from 2015 [45] and Model 2 used land derived using the automated classification of satellite
227 images from 2018 [53]. The list includes all predictor variables used in the development of the species-
228 specific ecological niche models and whether the variable was used in the final model.

| **Variable** | **Frequency** | **Final model** |
| --- | --- | --- |
| **Model 1:** | | |
| Native forest | yes | yes |
| Plantations of pine & eucalyptus | yes | yes |
| Wetlands | yes | yes |
| Pastures | yes | yes |
| Herbaceous crops of tobacco & maize | yes | yes |
| Shrub crops of tea & yerba mate | yes | yes |
| Mixed crops with small patches of shrubs & herbaceous plants | yes | yes |
| Naturally bare ground | yes | no |
| Water bodies (natural & artificial) | yes | no |
| Urban and infrastructure | yes | yes |
| Landscape heterogeneity index | yes | yes |
| Land use type | no | no |
| **Model 2:** | | |
| Native forest | yes | yes |
| Forest plantations | yes | yes |
| Wetlands | yes | yes |
| Pastures | yes | yes |

| Annual agricultural crops | yes | yes |
| --- | --- | --- |
| Mix of agriculture and pastures | yes | yes |
| No vegetation | yes | yes |
| Water | yes | no |
| Perennial agricultural crops | yes | yes |
| Landscape heterogeneity index | yes | yes |
| Land use type | no | no |

| 230 |  |
| --- | --- |
| 231 | The initial species-specific ecological niche models were run with all defined predictor variables, |
| 232 | 12 in Model 1 and 11 in Model 2 (Table 1). Evaluation of jackknife tests of variable importance and |
| 233 | response curves for individual variables eliminated select variables from each final model, due to either |
| 234 | no effect or a negative effect on model performance. Both models eliminated land use type and water. |
| 235 | Model 1 also eliminated bare ground. Interestingly, Model 2 maintained bare ground in the final |
| 236 | model; however, this classification appears more similar to the urban and infrastructure category in |
| 237 | Model 1. This evaluation used the regularized training gain and test gain generated by MaxEnt, which |
| 238 | accounts for dependency among predictor variables and compares the effect of a specific feature by |
| 239 | itself with a model of all features except that single feature. Efficacy of the nine selected predictor |
| 240 | variables were evaluated by MaxEnt jackknife tests using test gain and area under the ROC curve (AUC) |
| 241 | on test data, with the latter providing a threshold-independent measure of overall model accuracy |
| 242  243 | [84]. |
| 244 | **Potential species richness (PSR)** |

245 The PSR, which is obtained by combining the five species-specific ENMs, identifies areas where none
246 (value=0) or all (value=5) of the carnivores overlap in habitat defined as suitable [85]. PSR highlights
247 areas where multiple species have suitable habitat and quantifies species number. This degree of
248 overlap is an additional method to confirm range-restricted species and identify areas where habitat
249 restoration could result in increased PSR.

## 251 Identification of regions in need of management strategies

252 The five species-specific ENMs with suitable areas defined as marginal and optimal were combined to
253 generate a PSR and identify areas in need of management strategies. To simplify the analyses and
254 interpretation, data were grouped into six classes: 0 species or unsuitable habitat, 1-3 species with
255 marginal habitat in need of restoration, 4-5 species with marginal habitat in need of restoration, 1-3
256 species with optimal habitat in need of restoration and protection, 4 species with optimal habitat in
257 need of protection, and 5 species with optimal habitat in need of protection.

259	**Results**

## 260 Final ENM evaluation

261 Both models had AUC values that indicated a high accuracy in discriminating areas of species’
262 presence and absence [86]. In Model 1, the AUC values were ≥ 0.87 for test data (jaguar=0.96±0.01

263	(SD), puma=0.92±0.02, ocelot=0.89±0.02, southern tiger cat=0.87±0.01, bush dog=0.88±0.03). In

264	Model 2, the AUC values were ≥ 0.90 for test data (jaguar=0.96±0.01 (SD), puma=0.92±0.02,

265	ocelot=0.92±0.02, southern tiger cat=0.90±0.01, bush dog=0.91±0.02).

266 Each model was converted to a binary prediction of suitable and unsuitable habitat (Figure 2).
267 In Model 1, MTP best fits the defined threshold criteria in puma (0.0258), ocelot (0.0415), and southern
268 tiger cat (0.0055), while FCV1 was determined to be the best fit in jaguar (0.0145) and bush dog
269 (0.021). In Model 2, the results were similar, with MTP having the best fit for the defined threshold
270 criteria for puma (0.0376), ocelot (0.027), and southern tiger cat (0.00235), FCV1 was determined to be
271 the best fit in the jaguar (0.0234) and bush dog (0.0235). While FCV1 was determined to be the less
272 conservative choice in jaguar and bush dog for both models, it was considered the better choice
273 because it identified the maximum potential areas possible while still maintaining a zero-omission rate
274 for both training and test data.

### 276 Figure 2. A summary of the proportion (%) of marginal and optimal habitat defined as suitable in the

277 **northern-central zone for Model 1 (left) and Model 2 (right), with these two measures equal to the**
278 **binary prediction of proportion (%) of total suitable habitat.** Included are the five species-specific
279 ecological niche models and the potential species richness (PSR) that quantifies the overlap (0-5
280 species) among those areas defined as suitable for each carnivore. The latter is only represented as a
281 binary prediction.
283 When binary threshold values were assigned and the area was restricted to the northern-
284 central zone of Misiones (i.e., where the surveys occurred), the jaguar was identified as the most
285 restricted species (Table 2). However, there were differences among the models with Model 1 (57.7%)
286 having a higher total area defined as suitable habitat for the jaguar compared to either Model 2
287 (46.8%) or DeMatteo [1] (52.3%; Table 2). As with the jaguar, the other four carnivores had higher values in
288 Model 1 compared to DeMatteo [1]. While the ocelot had only a minimal difference between these
289 models (0.5%), there was a steady increase in this difference in puma (1.9%), southern tiger cat (3.6%),
290 jaguar (5.4%), and bush dog (12.5%; Table 2). In contrast, the pattern of Model 2 having a lower
291 proportion of total suitable habitat than Model 1 or DeMatteo [1] held true for southern tiger cat and
292 ocelot, while puma and bush dog had equal or higher proportions of total suitable habitat (Table 2).
293 When these values were averaged across the five carnivores, Model 1 had the highest average
294 proportion of suitable habitat (73.3%), while Model 2 (64.7%) fell below DeMatteo [1] (68.5%).

**Table 2. The proportion (%) of suitable habitat, as determined with species-specific ecological niche models (ENM), across the ~1.5M ha in the northern-central (N-C) zone in Misiones, Argentina, located outside of protected areas, and captured by ~400,000 ha multispecies biological corridor modelled by DeMatteo [1].** The N-C zone ALL refers to the proportion (%) of suitable habitat across the total area in the N-C zone of Misiones, including overlap with existing protected areas (~450,000 ha). In contrast, the N-C Zone OUTSIDE PA refers to the proportion (%) of total suitable habitat excluding areas that overlap with existing protected areas. The corridor refers to the proportion (%) of total suitable habitat captured by the DeMatteo [1] corridor. Each species has three suitability levels reported: total suitability, as defined by the binary threshold, and this area subdivided into marginal and optimal habitat. Each species has three ENMs reported: Model 1, Model 2, and the model generated by DeMatteo [1]. With the latter, no marginal or optimal habitat are available for comparison.

|  | **ENM** | **N-C zone ALL** | | | **N-C zone OUTSIDE PA** | | | **Corridor** | | |
| --- | --- | --- | --- | --- | --- | --- | --- | --- | --- | --- |
|  |  | Total | Marginal | Optimal | Total | Marginal | Optimal | Total | Marginal | Optimal |
| **Jaguar** | Model 1 | 57.7 | 32.5 | 25.2 | 30.2 | 18.9 | 11.3 | 33.0 | 18.9 | 14.1 |
|  | Model 2 | 46.8 | 33.3 | 13.5 | 21.8 | 17.3 | 4.5 | 55.3 | 42.3 | 13.0 |
|  | DeMatteo [1] | 52.3 | --- | --- | 25.4 | --- | --- | 13.9 | --- | --- |
| **Puma** | Model 1 | 68.4 | 31.7 | 36.7 | 39.9 | 24.51 | 15.4 | 32.6 | 17.4 | 15.2 |
|  | Model 2 | 66.5 | 39.5 | 27.0 | 38.5 | 27.3 | 11.2 | 35.3 | 21.4 | 13.9 |

|  | DeMatteo [1] | 66.5 | --- | --- | 37.6 | --- | --- | 21.0 | --- | --- |
| --- | --- | --- | --- | --- | --- | --- | --- | --- | --- | --- |
| **Ocelot** | Model 1 | 68.0 | 25.9 | 42.1 | 38.7 | 17.2 | 21.5 | 30.5 | 11.5 | 19.0 |
|  | Model 2 | 61.2 | 19.6 | 41.6 | 33.6 | 16.9 | 16.7 | 35.2 | 14.6 | 20.6 |
|  | DeMatteo [1] | 67.5 | --- | --- | 38.3 | --- | --- | 21.1 | --- | --- |
| **Southern tiger cat** | Model 1 | 85.9 | 47.3 | 38.6 | 56.6 | 33.7 | 22.9 | 28.1 | 9.8 | 18.3 |
|  | Model 2 | 70.1 | 22.4 | 47.7 | 42.4 | 19.1 | 23.3 | 33.8 | 10.4 | 23.4 |
|  | DeMatteo [1] | 82.3 | --- | --- | 52.9 | --- | --- | 24.3 | --- | --- |
| **Bush dog** | Model 1 | 86.6 | 52.9 | 33.7 | 58.6 | 37.3 | 21.3 | 29.7 | 13.9 | 15.8 |
|  | Model 2 | 78.8 | 54.1 | 24.7 | 51.6 | 33.7 | 17.9 | 30.9 | 16.9 | 14.0 |
|  | DeMatteo [1] | 74.1 | --- | --- | 45.4 | --- | --- | 22.5 | --- | --- |

Despite this range in the proportion of suitable habitat among the five carnivores in Model 1 (range=57.7-86.6%) and Model 2 (range = 46.8-78.8%), all had relatively high proportions of forest in their potential distributions, with only an ~11% spread or difference (Table 3). In both models, jaguar and ocelot had the highest proportions of forest, followed by puma, southern tiger cat, and bush dog. Like DeMatteo [1], puma, southern tiger cat, and bush dog had slightly higher levels of modified habitats than the jaguar and ocelot (Table 3). Breaking down these proportion of suitable habitat into the proportion located outside of protected areas allows insight into its sensitivity to ongoing changes in the landscape (Table 2). The average proportion across all five species showed little variation, with Model 1 the highest (44.8%) and Model 2 (37.6%) falling slightly below DeMatteo [1] (39.9%). Model 1 had a higher proportion of suitable area outside of protected areas in all species compared to Model 2 (Table 2). The jaguar had the lowest proportions of suitable habitat outside of protected areas (30.2% and 21.8%, respectively). The puma and ocelot were similar with slightly lower levels outside of protected areas, with Model 2 (33.6%) showing the ocelot as more restricted to protected areas. The southern tiger cat and bush dog had higher proportions of suitable habitat outside of protected areas (>50%), with the exception of Model 2 (42.4%) with the southern tiger cat.

When these binary thresholds of suitable habitat were further subdivided into marginal and optimal habitat (Figure. 2), the jaguar (10% and 14.3%) and bush dog (19.2% and 29.4%) had higher proportions of marginal habitat in both models, which contrasted with the ocelot (16.2% and 22%) that had higher proportions of optimal habitat in both models (Table 2). The puma and southern tiger cat showed a varied pattern, with the proportion of marginal and optimal switching between models (Table 2). When these proportion of marginal and optimal habitat were divided into the proportion located outside of protected areas, the jaguar, puma, and bush dog had higher portions of marginal

habitat located outside of protected areas, while the ocelot and southern tiger cat were varied in their proportions (Table 2).

## Potential species richness

These similarities and differences among the five carnivores in their proportion (Table 2) and type (Table 3) of suitable habitat help explain the PSR (Figure 2; Table 4). The five species-specific ENMs capture the overlap and unique characteristics of the five carnivores, as indicated by the finding that the proportion of area occupied by the highest PSR levels (≥4 species), with Model 1 (63.7%) having the highest proportion and Model 2 (57.3%) similar to DeMatteo [1] (59.4%; Table 4).

**Table 3. Proportion (%) of habitat relative to the total area defined as suitable in the species-specific ecological niche models (ENM) across the ~1.5M ha in the northern-central (N-C) zone in Misiones, Argentina.** Those values <1% are not reported. Each species has three ENMs reported: Model 1, Model 2, and the model generated by DeMatteo [1].

|  | **Model 1** | | | | | **Model 2** | | | | | | **DeMatteo [1]** | | | | | |
| --- | --- | --- | --- | --- | --- | --- | --- | --- | --- | --- | --- | --- | --- | --- | --- | --- | --- |
|  | Native forest | Plantations | Mixed crops | Shrub crops | Herbaceous crops | Native forest | Forest plantations | Pastures | Annual agriculture crops | Mix of agriculture & pastures | Perennial agricultural crops | Forest | Tree plantations | Agriculture | Mixed use | Urban | Pasture |
| **Jaguar** | 89.4 | 6.3 | 1.6 | 1.3 | 1.2 | 89.4 | 6.7 | 1.3 | --- | --- | 1.5 | 77.2 | 9.9 | 5.6 | 2.5 | 2.1 | --- |
| **Puma** | 85.8 | 6.9 | 3.1 | 2.4 | 1.7 | 82.7 | 6.9 | --- | 2.2 | 3.6 | 3.2 | 74.4 | 9.6 | 8.5 | 3.2 | 1.6 | 1.0 |
| **Ocelot** | 87.6 | 5.2 | 2.4 | 2.2 | 1.8 | 87.1 | 4.3 | --- | 1.7 | 2.8 | 3.0 | 79.8 | 8.7 | 4.2 | 3.5 | 1.3 | 1.1 |
| **Southern tiger cat** | 78.8 | 8.3 | 4.8 | 4.7 | 2.6 | 81.8 | 7.4 | --- | 2.3 | 4.0 | 3.6 | 70.7 | 10.8 | 8.3 | 5.5 | 1.9 | 1.7 |
| **Bush dog** | 78.1 | 5.9 | 6.8 | 5.1 | 3.8 | 78.6 | 5.6 | --- | 3.6 | 6.6 | 4.6 | 75.1 | 7.3 | 7.7 | 6.1 | --- | 2.2 |

**Table 4. The proportion (%) of suitable habitat across the range of potential species richness (0-5 species) within the ~1.5M ha of the northern-central (N-C) zone in Misiones, Argentina, as well as the proportion (%) of total suitable habitat (0-5 species) captured by ~400,000 ha multispecies biological corridor modelled by DeMatteo [1].** Each species has three ENMs compared: Model 1, Model 2, and the model generated by DeMatteo [1].

|  | **Model 1** | | **Model 2** | | **DeMatteo [1]** | |
| --- | --- | --- | --- | --- | --- | --- |
|  | **N-C zone** | **Corridor** | **N-C zone** | **Corridor** | **N-C zone** | **Corridor** |
| **0 species** | 4.5 | --- | 12.8 | --- | 7.8 | --- |
| **1 species** | 14.3 | 1.8 | 14.5 | 1.4 | 13.9 | 2.3 |
| **2 species** | 8.6 | 1.3 | 7.4 | 1.6 | 9.8 | 2.4 |
| **3 species** | 8.9 | 2.4 | 8.0 | 2.3 | 9.1 | 2.9 |
| **4 species** | 10.2 | 3.0 | 16.3 | 8 | 15.3 | 6.1 |
| **5 species** | 53.5 | 18.8 | 41.0 | 15.9 | 44.1 | 14.3 |

## Evaluation of DeMatteo [1] corridor

The proportion of suitable habitat captured by the DeMatteo [1] corridor can allow insight into the risk still facing each carnivore, as these areas represent regions lacking formal protection, with all areas intersecting protected areas excluded from these analyses. (Table 2). Model 2 was slightly higher than Model 1 in the proportion of suitable habitat (1.2-5.7%) captured by the corridor, with puma, ocelot, southern tiger cat, and bush dog (Table 2). The exception to this pattern was the jaguar that had a large increase (22.3%) in the proportion of suitable habitat captured by the corridor in Model 2

(Table 2). This trend holds both models compared to the original model, with Model 2 (8.4-41.4%)

having a larger proportion of suitable habitat captured by the corridor across all five species than Model 1 (3.8-19.1%; Table 2). Both Model 1 (30.8%) and Model 2 (38.1%) captured a higher average proportion of suitable habitat compared to DeMatteo [1] (20.6%), with Model 2 capturing almost 2x the average proportion of the original model.

The proportion of marginal and optimal habitat captured by the DeMatteo [1] corridor was similar for the bush dog (Table 2). The ocelot and southern tiger cat showed a higher proportion of optimal habitat in the corridor, while the jaguar and puma were the opposite with a higher proportion of marginal habitat captured by the corridor (Table 2). Even with these differences, the spread or difference between the proportion of optimal and marginal habitat captured by the corridor were relatively low in Model 1 (1.9-8.5%) and Model 2 (2.9-7.5%), with the exception of the jaguar (+29.3%) and southern tiger cat (+13.0%), where more marginal habitat was captured in Model 2 (Table 2).

The proportion of the highest PSR levels (≥4 species) captured by the DeMatteo [1] corridor was similar when compared to each other (21.8% and 23.9%) or the original model (20.4%) (Table 4).

However, when only the top level of PSR (5 species) captured by the corridor was examined, both Model 1 (18.8%) and Model 2 (15.9%) had higher proportions of suitable habitat than DeMatteo [1]

(14.3%)

As with DeMatteo [1], both models had higher levels of native forest (79.1% and 75.6%) versus modified habitats occupying the corridor (Table 5), both models having proportions higher than the original (61.9%). The high proportion of native forest occupying the corridor is similar to the proportions of native forest across the five ENMs in the northern-central zone for both models (84.0% and 83.9%), with values again being higher than the original model (75.4%; Table 5). While land use defined categories varied according to the data used in the models, modified land use types grouped into three main categories: forest plantations, agriculture at varying scales, and pastures.

**Table 5. Proportion (%) of native forest and modified environments for the northern-central (NC) zone in Misiones, Argentina, and the ~400,000 ha multispecies biological corridor modelled by DeMatteo [1].** Those values <1% are not reported. Each species has three ENMs compared: Model 1, Model 2, and the model generated by DeMatteo [1].

| **Variable** | **N-C zone** | **Corridor** |
| --- | --- | --- |
| **Model 1:** | | |
| Native forest | 72.3 | 79.1 |
| Plantations of pine & eucalyptus | 8.9 | 5.6 |
| Wetlands | --- | --- |
| Pastures | --- | --- |
| Herbaceous crops of tobacco & maize | 3.9 | 3.7 |
| Shrub crops of tea & yerba mate | 7.0 | 5.4 |
| Mixed crops with small patches shrubs & herbaceous plants | 7.0 | 6.1 |
| Naturally bare ground | --- | --- |
| Water bodies (natural & artificial) | --- | --- |
| Urban and infrastructure | --- | --- |
| **Model 2:** | | |
| Native forest | 71 | 75.6 |
| Forest plantations | 10.5 | 8.0 |
| Wetlands | --- | --- |

| Pastures | --- | --- |
| --- | --- | --- |
| Annual agricultural crops | 3.4 | 3.5 |
| Mix of agriculture and pastures | 7.2 | 6.6 |
| No vegetation | 1.0 | --- |
| Water | --- | --- |
| Perennial agricultural crops | 6.1 | 5.6 |
| **DeMatteo [1]** | | |
| Forest | 61.9 | 61.9 |
| Tree plantations | 13.0 | 14.1 |
| Agriculture | 10.7 | 10.1 |
| Mixed use | 6.1 | 7.0 |
| Pasture | 3.0 | 2.4 |
| Bare ground | 1.9 | 2.3 |
| Water | 0.5 | --- |
| Urban | 1.0 | 0.4 |
| Campos/grasslands | --- | --- |
| Unclassified | 1.9 | 1.8 |

## Identification of regions in need of management strategies

To evaluate the areas of the corridor that need habitat restoration and/or habitat protection, the levels of PSR, as defined by marginal and optimal habitat, were evaluated (Figure 3). This refined analysis goes beyond DeMatteo [1], with the ability to evaluate the quality of habitat for these overlapping

species. Across the northern-central zone, both models capture similar totals of suitable habitat (27.7% and 29.3%) and have similar levels of marginal habitat (31.6% and 26.7%), optimal habitat with 1-3 species (44.2% and 45.7%), and optimal habitat with 4-5 species (19.7% and 14.8%) (Table 6). In addition, both models have similar levels of marginal habitat captured in the DeMatteo [1] corridor (19.7% and 17.6%) (Table 6); however, there are slight differences in the proportions of optimal habitat that are contained within the corridor of DeMatteo [1]. The levels of Model 1 are more similar in distribution of optimal habitat with 1-3 species (46.0%) and 4-5 species (34.0%), while Model 2 which has a higher proportion of optimal habitat with 1-3 species (62.3%) compared to 4-5 species (18.2%) (Table 6).

### Figure 3. The potential species richness (PSR) represented as a summary of the proportion (%) of marginal and optimal habitat defined as suitable in the northern-central zone for Model 1 and Model

**2.** For each model, the overlap of marginal and optimal habitats was grouped into six classes: 0 species, 1-3 species with marginal habitat, 4-5 species with marginal habitat, 1-3 species with optimal habitat, 4 species with optimal habitat, and 5 species with optimal habitat. Each level is then categorized into the type of land management that is needed to optimized long-term survival of species in the corridor: habitat restoration, habitat protection, or a combination of the two practices.

**Table 6. The potential species richness (PSR) with habitat suitability subdivided into marginal and optimal habitat.** For each model two summaries are reported: 1) the proportion (%) of suitable habitat across the range of PSR within the ~1.5M ha of the northern-central (N-C) zone in Misiones, Argentina and 2) the proportion (%) of PSR found within ~400,000 ha multispecies biological corridor modelled by DeMatteo [1]. While marginal habitat is exclusive, within optimal habitat, there are varying levels of

marginal habitat that overlap but simplify the categories, only the number of species for optimal habitat are reported.

|  |  | **Model 1** | | **Model 2** | |
| --- | --- | --- | --- | --- | --- |
| **# Species** | **Habitat type** | **N-C zone** | **Corridor** | **N-C zone** | **Corridor** |
| **0** | **unsuitable** | 4.5 | 0.3 | 12.8 | 1.9 |
| **1-3** | **marginal** | 26.8 | 14.6 | 23.3 | 12.0 |
| **4-5** | **marginal** | 4.8 | 5.1 | 3.4 | 5.6 |
| **1-3** | **optimal** | 44.2 | 46.0 | 45.7 | 62.3 |
| **4** | **optimal** | 11.2 | 15.9 | 10.5 | 9.6 |
| **5** | **optimal** | 8.5 | 18.1 | 4.3 | 8.6 |

Within the DeMatteo [1], corridor connectors were defined as those regions with the lowest level of PSR and habitat integrity. These regions occupy ~30% of the corridor and were identified as areas that put connectivity between the northern-central zone at risk. This reanalysis with suitable habitat subdivided into marginal and optimal habitat highlights that not all areas within the connector are equal in their needs (Figure 4 and Table 6). While both models require restoration of the marginal habitat that makes up about half of the connectors (58.4% and 51.0%), both models also have a large portion that need a combined management approach. While Model 2 has the majority requiring restoration plus protection (48.4%), Model 1 has 35.4% in this category and 6.2% in the category of needing protection with optimal habitat and high PSR.

### Figure 4. The potential species richness (PSR) is represented as a summary of the proportion (%) of marginal and optimal habitat defined as suitable in the northern-central zone for Model 1 and Model

**2. Presented is the overlap of the potential species richness (PSR) with defined marginal and optimal habitats for each model with the DeMatteo [1] corridor, specifically the connectors or those areas defined as having the lowest levels of PSR and habitat integrity.** Areas in the connectors are defined by the type of habitat (marginal or optimal), the number of overlapping species (1-5), and whether habitat restoration and/or protection are needed. The remainder of the corridor is generalized as the core buffer, as it corresponds to the DeMatteo [1] model.

# Discussion

This study used a downstream, multispecies approach where five carnivores, with similar but disparate ecological sensitivities to disturbance, were evaluated individually and then overlapped to identify common priority areas [38]. As with DeMatteo [1], this expansion beyond a single species approach captured the varied ecological requirements of coexisting carnivores versus the limited requirements of the most restricted species (i.e., jaguar; Figure 1 and Table 2). While the five carnivores vary in their body size, there is a strong intersection in their ecological requirements, with a 41-53.5% (Table 4) overlap in suitable habitat among the five in the northern-central zone. The fact that this overlap is at most ~50% makes it clear that there is existing variation among the five carnivores in the degree of habitat flexibility or sensitivity to human disturbance [20,51,52,87,88]. By developing species-specific ENMs that incorporated frequency of intact or altered habitat (Table 1) in a corresponding area of use, it was possible to quantify the area defined as suitable for each species (Table 2) and differentiate the factors that constrained this suitability (Table 3).

While the two models differed in the proportions of suitable habitat and associated vegetation, when compared to each other and DeMatteo [1], the general patterns were consistent across the five carnivores. In contrast to the highly-restricted jaguar, the southern tiger cat and the bush dog had the largest suitable areas in the northern-central zone of Misiones, followed by the ocelot and puma (Figure 2). A similar pattern is seen among the five carnivores in the proportion of suitable habitat outside of protected areas, which are primarily composed of native forest, with the southern tiger cat and bush dog almost 2x that of the jaguar (Table 2). The jaguar’s low proportion of suitable habitat outside of protected areas (21.8-30.2%) provides insight into its preference to proximity to large tracts of forest versus altered habitat. Similarly, the puma and ocelot are associated with higher levels of forest, which explains the slightly lower levels of suitable habitat for these species in the northern- central zone and outside of protected areas compared to the levels found in the southern tiger cat and bush dog.

When these levels of suitability are subdivided, it is the two species at the extremes in the proportion of suitable habitat, jaguar and bush dog, that have the highest proportions of marginal habitat in the northern-central zone. This is compared to the moderate ocelot that has higher proportions of optimal habitat. When narrowed to the areas outside of protected areas, the jaguar and bush dog are joined by the puma in sharing the highest levels of marginal habitat. Together these findings suggest that the long-term survival of the jaguar in the northern-central zone is at risk with areas outside of protected areas of only marginal habitat quality. While the bush dog appears to be more flexible, it is critical to remember that while it is a species that exists across varied habitats, these habitats typically provide lower quantities of prey and a higher risk for exposure to domestic dog diseases [81,89]. The same is true for the puma, which is a high risk of poaching when crossing open areas near human- occupied areas. Interestingly, the ocelot, which is associated with higher levels of forest in this study

and others [88,90], still shows a degree of flexibility outside of protected areas with high levels of optimal habitat, which suggests it might not be as restricted in the variety of habitats it can occupy [91].

Using species-specific ENMs, DeMatteo [1] developed resistance layers for each carnivore, which when merged provided a composite layer of potential movement across the landscape [38]. Using this combined resistance map for the five species, DeMatteo [1] centered the ~400,000 ha corridor on areas with the lowest ecological cost for the five carnivores. However, unlike DeMatteo [1] where the jaguar was the most restricted, as reflected by the lowest proportion (13.9%) of suitable habitat in the corridor compared to the other four carnivores (21.0-24.3%), the current models show similar proportions of suitable habitat contained within the corridor for each of the five carnivores (Table 2). Both models capture a higher average proportion of suitable habitat (1.5-2x) across the carnivores compared to DeMatteo [1]. While the habitat flexibility of the bush dog and puma are

reflected in the higher proportions of marginal habitat contained in the corridor, the risk to the jaguar’s mobility between protected areas is echoed with its higher proportion of marginal habitat. The ocelot’s comparatively higher level of optimal habitat in the corridor, again puts into question, its potential habitat flexibility [91].

Given that the DeMatteo [1] corridor connects existing suitable habitats in a heterogeneous landscape versus man-made structures as connectors, an investment to conserve this ~400,000 ha area would be considered worthwhile [92]. However, the process to determine key conservation targets must balance multiple factors, including species richness and cost allocation [93], which inevitably overlaps with finding a balance between the contrasting needs of wildlife and humans [94,95,96,97,98], especially as humans continue to expand into the backyards of wildlife [99,100,101,102]. While DeMatteo [1] used overlapping habitat suitability and degree of habitat fragmentation to differentiate areas that need varying degrees of protection and habitat restoration,

these analyses expanded beyond general suitability and into varying levels of suitability. While both models were similar in the proportion of area needing restoration (high PSR with marginal habitat), a combined approach of restoration-protection (low PSR with optimal habitat), or protection (high PSR with optimal habitat), this refined analysis highlighted some key differences (Table 6). Specifically, when focused on the areas defined as connectors or those areas with the lowest level of PSR and habitat integrity [1]. While these refined analyses found that ~50% of the connectors were composed of marginal habitat, the remainder corresponds to optimal habitat with varying levels of PSR (Figure 4 and Table 6). This means that management in these connectors must include work with the existing landowners (103,104,105) and extend beyond restoration and include protection of the optimal habitat and the species that occupy it. These results suggest there remains a high degree of connection between the northern-central zones of Misiones but without immediate action to protect the optimal habitat in the eastern portion of the connector, the varying levels of PSR are put at risk.

One question that remains is which of the two models more accurately reflects the situation in northern-central Misiones and the corridor of DeMatteo [1]. While one might argue that Model 1 (2015) is based on older data where higher levels of native forest would exist. An initial comparison of the habitat associated with individual scats between the two years supports this idea with all five species having lower levels of forest in Model 2 compared to Model 1, with similar levels in the four felids (84.7-89.1), but lower levels in the bush dog. However, further analyses that examine other types of altered habitat suggests that the difference in years does not explain all the differences.

Instead, there appears to be a discrepancy in how the pixels are defined versus an actual shift in habitat quality (e.g., intact versus altered). For example, with the southern tiger cat, Model 2 aligns with only 64.1% forestry plantations, with the remaining proportion (35.9%) including areas with no

vegetation, forest, and annual agriculture crops. These differences expand with other habitat types, with Model 2 aligning with only 16.2% shrub crops, 6.7% herbaceous crops, and 5% mixed use of Model 1, with the remaining portion including a mix of native forest and other types of altered habitats. The terrain in Misiones outside of protected areas is extremely heterogeneous with a mix of habitat types in close proximity to each other. What was historically shrub crops of only yerba, are now frequently a mix of yerba planted among a network of native trees. While some pine plantations are composed of densely planted trees, others are more widely spaced allowing a native forest to become established. In some cases, additional ground truthing for defined areas of different vegetation types may help but in others the overlap between pixel values may be blurred making a clean definition of one type or the other impossible or varied between interpretations. Despite the differences in the individual layers used in each model, the final results were comparable, suggesting the definition of intact or altered is consistent even if the defined type of habitat varies.

While this model was comprehensive with a multispecies approach that incorporated anthropogenic variation across the landscape [38], future models could expand in several directions. First, the Green Corridor of Misiones expands into the central-southern zone of Misiones. While climatic conditions are similar in this region, the degree of ongoing habitat conversion, poaching, and growing populations are magnitudes higher. Understanding how species distributions shift in their unique and overlapping values is essential to developing conservation strategies in this region. This type of analysis can also extend outside of Misiones, as the species in this study have ranges across the Neotropics. Therefore, the results from this study can be expanded to understand the connectivity between countries, with analyses at the species or community level [106]. Second, analyses could expand to include other variables, such as the overlap of prey species and presence of illegal hunting. Both of these factors directly and indirectly affect the distribution of carnivores. Finally, these types of

analyses could explore how future climate predictions could shift the distribution of these five carnivores [107,108,109,110]. While there northern-central zones of Misiones show little variation in climate, it is evident that what was once considered ‘normal’ is shifting. The range of seasonality in both temperatures and rainfall is more variable, leading to shifts in water availability, soil moisture levels, and risk of fire. Given the distribution of these five carnivores beyond the boundaries of Misiones and the ever-expanding human footprint [2,3], there is a need to understand how ongoing habitat fragmentation and shifts in climate can affect the long-term survival of these species.

# Acknowledgments

The Ministerio de Ecología y Recursos Naturales Renovables of Misiones (MEyRNR) and the Administración de Parques Nacionales of Argentina provided permits and help with field planning. The MEyRNR provided housing. Multiple US zoos (Birmingham Zoo, Cincinnati Zoo, Detroit Zoo, Exotic Feline Breeding Compound, Little Rock Zoo, Palm Beach Zoo, Saint Louis Zoo, San Antonio Zoo, and Sedgwick County Zoo) and three field researchers (C. Vynne, C. Wultsch, and N. Songsasen) provided detection dog training samples. Sincere thanks to the numerous Argentinean students who assisted in the field, the provincial/national park guards, NGO’s, local conservationists, private land/reserve owners, and colleagues that helped with various aspects of this project in the field and in the lab.

Thank you to PackLeader Conservation Detection Dogs for guidance and to all members of the Olsen/Eggert Labs for their support. Of course, a special thanks to “T” whose amazing work ethic made it all possible.

# References

1. DeMatteo KE, Rinas MA, Zurano JP, Selleski N, Schneider RG, Argüelles CF. Using niche-modelling and species-specific cost analyses to determine a multispecies corridor in a fragmented landscape. PLoS ONE. 2017;12(8):e0183648. http://journals.plos.org/plosone/article?id=10.1371/journal.pone.0183648
2. Venter O, Sanderson EW, Magrach A, Allan JR, Beher J, Jones KR, et al. Global terrestrial Human Footprint maps for 1993 and 2009. Sci Data. 2016;3:160067. DOI: 10.1038/sdata.2016.67
3. Venter O, Sanderson EW, Magrach A, Allan JR, Beher J, Jones KR, et al. Sixteen years of change in the global terrestrial human footprint and implications for biodiversity conservation. Nat Commun. 2016;7:12558. DOI:10.1038/ncomms1255.
4. McShane TO, Hirsch PD, Trung TC, Songorwa AN, Kinzig A, Monteferri B, et al. Hard choices: making trade-offs between biodiversity conservation and human well-being. Biol. Conserv. 2011;144:966- 972.
5. Winn MI, Pogutz S. Business, ecosystems, and biodiversity: new horizons for management research. Organ Environ. 2013;26(2):203-229.
6. Laurila-Pant M, Lehikoinen A, Urusitalo L, Venesjärvi R. How to value biodiversity in environmental management? Ecol Indic. 2015;55:1-11.
7. Barrett CB, Travis AJ, Dasgupta P. On biodiversity conservation and poverty traps. PNAS.

2011;108(34):13907-13912.

8. Mace GM, Norris K, Fitter AH. Biodiversity and ecosystem services: a multilayered relationship.

TREE. 2012;27(1):19-26.

9. Schneiders A, Van Daele T, Van Landuyt W, Van Reeth W. Biodiversity and ecosystem services: complementary approaches for ecosystem management? Ecol Indic. 2012;21:123-33.
10. Carwardine J, O’Connor T, Legge S, Mackey B, Possinghan HP, Martin TG. Prioritizing threat management for biodiversity conservation. Conser Lett. 2012;5:196-204.
11. Ray N, Lehmann A, Joly P. Modelling spatial distribution of amphibian populations: a GIS approach based on habitat matrix permeability. Biodivers Conserv. 2002;11:2143-2165.
12. Nikolakaki P. A GIS site-selection process for habitat creation: estimating connectivity of habitat patches. Landsc Urban Plan. 2004;68:77-94.
13. Bruner AG, Gullison RE, Balmford A. Financial costs and shortfalls of managing and expanding protected-areas systems in developing countries. Biosci. 2004;54:1119-1126.
14. Schmidt-Soltau K, Brockington D. Protected areas and resettlement: what scope for voluntary relocation? World Dev. 2007;35:2182-2202.
15. DeFries R, Karanth KK, Pareeth S. Interactions between protected areas and their surroundings in human-dominated tropical landscapes. Biol Conserv. 2010;143:2870-2880.
16. Aichi Biodiversity Targets. Aichi Biodiversity Targets from the Convention on Biological Diversity - Strategic Plan for Biodiversity 2011-2020. Available from: <https://www.cbd.int/sp/targets/>.
17. Santini L, Santiago S, Rondinini C. Connectivity of the global network of protected areas. Divers Distrib. 2016;22:199-211.
18. Fuller R, Munguía M, Mayfield M, Sánchez-Cordero V, Sarkar S. Incorporating connectivity into conservation planning: a multi-criteria case study from central Mexico. Biol Conserv. 2006;133:131- 142.
19. De Angelo C, Paviolo A, Di Bitetti MS. Differential impact of landscape transformation on pumas (*Puma concolor*) and jaguars (*Panthera onca*) in the Upper Paraná Atlantic Forest. Divers Distrib. 2011;17:422-436.
20. DeMatteo KE, Rinas MA, Argüelles CF, Holman BE, Di Bitetti MS, Davenport B, et al. Using detection dogs and genetic analyses of scat to expand knowledge and assist felid conservation in Misiones, Argentina. Integr Zool. 2014;9:623-639.
21. Beier P, Majka DR, Spencer WD. Forks in the road: choices in procedures for designing wildland linkages. Conserv Biol. 2008;22:836-851.
22. Rayfield B, Fortin M-J, Fall A. The sensitivity of least-cost habitat graphs to relative cost surface values. Landsc Ecol. 2010;25:519-522.
23. Boyce MS, Vernier PR, Nielsen SE, Schmiegelow FKA. Evaluating resource selection functions. Ecol Modell. 2002;157:281-300.
24. Chetkiewics CLB, Boyce MS. Use of resource selection functions to identify conservation corridors. J Appl Ecol. 2009;46:1036-1047.
25. Long RA, Muir JD, Rachlow JI, Kie JG. A comparison of two modeling approaches for evaluating wildlife-habitat relationships. J Wildl Manage. 2009;73(2):294-302.
26. Rabinowitz A, Zeller KA. A range-wide model of landscape connectivity and conservation for the jaguar, *Panthera onca*. Biol Conserv. 2010;143:939-945.
27. Dixon JD, Oli MK, Wooten MC, Eason TH, McCown JW, Paetkau D. Effectiveness of a regional corridor in connecting two Florida black bear populations. Conserv Biol. 2006;20:155-162.
28. Cascelli de Azevedo FC, Murray DL. Spatial organization and food habits of jaguars (*Panthera onca*) in a floodplain forest. Biol Conserv. 2007;137:391-402.
29. Vynne C, Keim JL, Machado RB, Marinho-Filho J, Silveira L, Groom MJ, et al. Resource selection and its implications for wide-ranging mammals of the Brazilian cerrado. PLoS ONE. 2011;6(12):e28939. doi: 10.1371/journal.pone.0028939.
30. McRae BH, Beier P. Circuit theory predicts gene flow in plant and animal populations. Proc Natl Acad Sci U S A. 2007;104:19885-19890.
31. Phillips SJ, Williams P, Midgley G, Archer A. Optimizing dispersal corridors for the Cape Proteaceae using network flow. Ecol Appl. 2008;18:1200-1211.
32. Beier P, Majka DR, Newell SL. Uncertainty analysis of least-cost modelling for designing wildlife linkages. Ecol Appl. 2009;19:2067-2077.
33. Cushman SA, McKelvey KS, Schwartz MK. Use of empirically derived source-destination models to map regional conservation corridors. Conserv Biol. 2009;23:368-376.
34. Parks SA, McKelvey KS, Schwartz MK. Effects of weighting schemes on the identification of wildlife corridors generated with least-cost methods. Conserv Biol. 2013;27:145-154.
35. Carvalho F, Carvalho R, Mira A, Beja P. Assessing landscape functional connectivity in a forest carnivore using path selection functions. Landsc Ecol. 2016;31:1021-1036.
36. Sawyer SC, Epps CW, Brashares JS. Placing linkages among fragmented habitats: do least-cost models reflect how animals use landscapes? J Appl Ecol. 2011;48:668-678.
37. Brodie JF, Giordana AJ, Dickson B, Hebblewhite M, Bernard H, Mohd-Azlan J, et al. Evaluating multispecies landscape connectivity in a threatened tropical mammal community. Conserv Biol. 2014;29:122-132.
38. Wood SLR, Martins KT, Dumais-Lalonde V, Tangua O, Maure F, St-Denis A, et al. Missing interactions: the current state of multispecies connectivity analysis. Front Ecol Evol. 2022;10: 10.830822. doi: 103389/fevo.2022.830822.
39. Lambeck RJ. Focal species: a multi-species umbrella for nature conservation. Conserv Biol.

1997;11:849-856.

40. Simberloff D. Flagships, umbrellas, and keystones: is single species management passe in the landscape era? Biol Conserv. 1998;83:247-257.
41. Brost BM, Beier P. Comparing linkage designs on land facets to linkage designs based on focal species. PLoS ONE. 2012;7(11):848965. doi: 10.137/journal.pone.0048965.
42. Krosby M, Breckheimer I, Pierce DJ, Singleton PH, Hall SA, Halupka KC, et al. Focal species and landscape “naturalness” corridor models offer complementary approaches for connectivity conservation planning. Landsc Ecol. 2015;30:2121-2132.
43. Meyers N, Mittermeier RA, Mittermeier CG, Da Fonseca GA, Kent J. Biodiversity hotspots for conservation priorities. Nature. 2000;403(6772):853-858.
44. Hrdina M, Romportl D. Evaluating global biodiversity hotspots – very rich and even more endangered. J Landsc Ecol. 2017;10(1):108-115.
45. Zuleta GA, Gauto OA, Varela DM, De Angelo C, Johnson BG, Lorán D, et al. Evaluaciones Ambientales Estratégicas y Programa de Monitoreo de la Biodiversidad en las Regiones de Mesopotamia y Delta de Paraná. Final Report (Informe Final). 2016. Ministerio de Agroindustria de la Nación. Univ. Maimónides-Univ. Nac. De Misiones-CONICET, Argentina.
46. Paviolo A, De Angelo CD, Di Blanco YE, Di Bitetti MS. Jaguar *Panthera onca* population decline in the Upper Paraná Atlantic Forest of Argentina and Brazil. Oryx. 2008;42:554-561.
47. Paviolo A, Di Blanco YE, De Angelo CD, Di Bitetti MS. Protection affects puma abundance and activity patterns in the Atlantic Forest. J Mammal. 2009;90:926-934.
48. Haag T, Santos AS, Sana DA, Morato RG, Cullen Jr, L, Crawshaw Jr PG, et al. The effect of habitat fragmentation on the genetic structure of a top predator: loss of diversity and high differentiation among remnant populations of Atlantic Forest jaguars (*Panthera onca*). Mol Ecol. 2010;19:4906- 4921.
49. Iezzi ME, Cruz P, Varela D, De Angelo C, Di Bitetti MS. Tree monocultures in a Biodiversity hotspot: impact of pine plantations on mammal and bird assemblages in the Atlantic Forest. For Ecol Manag. 2018;424:216-227.
50. Sancha NU, Higgins CL, Presley SJ, Strauss RE. Metacommunity structure in a highly fragmented forest: has deforestation in the Atlantic Forest altered historic biogeographic patterns? Divers Distrib. 2014;20:1058-1070.
51. DeMatteo KE, Rinas MA, Argüelles CF, Zurano JP, Selleski N, Di Bitetti MS, et al. Noninvasive techniques provide novel insight for the elusive bush dog (*Speothos venaticus*). Wildl Soc Bull. 2014;38(4):862-873.
52. Izquierdo AE, De Angelo CD, Aide TM. Thirty years of human demography and land-use change in the Atlantic Forest of Misiones, Argentina: an evaluation of the forest transition model. Ecol Soc. 2008;13:3. [online] URL: <http://www.ecologyandsociety.org/vol13/iss2/art3/>.
53. MapBiomas Bosque Atlántico Trinacional – Collection 1 of Atlantic Forest Annual Land Cover & Land Use Map Series, accessed on 11/2020 through the link: [https://mapbiomas.org](https://mapbiomas.org/).
54. Kiesecker JM, Copeland H, Pocewicz A, McKenney B. Development by design: blending landscape- level planning with the mitigation hierarchy. Front Ecol Environ. 2010; 8(5):261-266.
55. Keesstra S, Nunes J, Novara A, Finger D, Avelar D, Kalantari C. The superior effect of nature based solutions in land management for enhancing ecosystem services. Sci Total Environ. 2018;610-611: 997-1009.
56. Sandker M, Campbell BM, Ruiz-Pérez M, Sayer JA, Cowling R, Habtemariam K, et al. The role of participatory modeling in landscape approaches to reconcile conservation and development. Ecol Soc. 2010;15(2):13. URL: [http://www.ecologyandsociety.org/vol15/iss2/art13.](http://www.ecologyandsociety.org/vol15/iss2/art13)
57. Young JC, Jordan A, Searle KR, Butler A, Chapman DS, Simmons P, et al. Does stakeholder involvement really benefit biodiversity conservation. Biol. Conserv. 2013;158:359-370.
58. Young JC, Jordan A, Searle KR, Butler A, Simmons P, Watt AD. Framing scale in participatory biodiversity management may contribute to more sustainable solutions. Conserv Lett. 2013;6(5):333-340.
59. Boiral O, Heras-Saizarbitoria I. Managing biodiversity through stakeholder involvement: why, who, and what initiatives? J Bus Ethics 2017;140:403-421.
60. DeMatteo KE, Rinas MA, Sede MM, Davenport B, Argüelles CF, Lovett K, et al. Detection dogs: an effective technique for bush dog surveys. J Wildl Manage. 2009;73:1436-1440.
61. Vynne C. Landscape use by wide-ranging mammals of the Brazilian Cerrado. Ph.D. Dissertation, University of Washington, Seattle, Washington. 2010. Available from: <https://search.proquest.com/docview/816087316>
62. Farrell LE, Roman J, Sunquist ME. Dietary separation of sympatric carnivores identified by molecular analysis of scats. Mol Ecol. 2000;9:1583–90.
63. Miotto RA, Rodrigues FP, Ciocheti G, Galetti Jr PM. Determination of the minimum population size of pumas (*Puma concolor*) through fecal DNA analysis in two protected cerrado areas in the Brazilian southeast. Biotropica. 2007;39:647–54.
64. Altschul SF, Gish W, Miller W, Myers EW, Lopman DJ. Basic local alignment search tool. J Mol Biol.

1990;215:403-410.

65. Phillips SJ, Anderson RP, Schapire RE. Maximum entropy modeling of species geographic distributions. Ecol Modell. 2006;190:231-259.
66. Elith, J, Graham CH, Anderson RP, Dudík M, Ferrier S, Guisan A, et al. Novel methods improve prediction of species’ distributions from occurrence data. Ecography. 2006;29:129-151.
67. Duan R-Y, Kong X-Q, Huang M-Y, Fan W-Y, Wang Z-G. The predictive performance and stability of six species distribution models. PLoS ONE. 2014;9(11):e112764. doi: 10.1371/journal.pone.0112764
68. Phillips SJ, Dudík M. Modeling of species distributions with Maxent: new extensions and a comprehensive evaluation. Ecography. 2008;31:161-175.
69. Merow C, Smith MJ, Silander JA. A practical guide to MaxEnt for modeling species’ distributions.

What it does, and why inputs and settings matter. Ecography. 2013;36:1058-1069.

70. Liu C, Berry PM, Dawson TP, Pearson RG. Selecting thresholds of occurrence in the prediction of species distributions. Ecography. 2005;28:385-393.
71. Trisurat Y, Bhumpakphan N, Reed DH, Kanchanasaka B. Using species distribution modeling to set management priorities for mammals in northern Thailand. J Nat Conserv. 2012;20:264-273.
72. Liu C, White M, Newell G, Pearson R. Selecting thresholds for the prediction of species occurrence with presence-only data. J Biogeogr. 2013;40:778-789.
73. Liu C, Newell G, White M. On the selection of thresholds for predicting species occurrence with presence-only data. Ecol Evol. 2016;6:337-348.
74. Izquierdo AE, Clark ML. Spatial analysis of conservation priorities based on ecosystem services in the Atlantic forest region of Misiones, Argentina. Forests. 2012;3:764-786.
75. Hirzel AH, Hausser J, Chessel D, Perrin N. Ecological-niche-factor analysis: how to computer habitat- suitability maps without absences data? Ecology. 2002;83:2027-2036.
76. Crawshaw PG, Jr. Comparative ecology of ocelot *Felis pardalis* and jaguar *Panthera once* in a protected subtropical forest in Brazil and Argentina. Ph.D. Dissertation, University of Florida, Gainesville. 1995.
77. Cullen L Jr, Abreu KC, Sana D, Nava AFD. Jaguars as landscape detectives for the upper Paraná River corridor, Brazil. Nat Conserv. 2005;3:43-58.
78. Astete S, Sollmann R, Silveira L. Comparative ecology of jaguars in Brazil. Cat News. 2008;4:9-14.
79. Gonzalez-Borrajo N, López-Bao JV, Paomares F. Spatial ecology of jaguars, pumas, and ocelots: a review of the state of knowledge. Mammal Rev. 2017;47:62-75.
80. Sunquist M, Sunquist F. Puma. In: Wild Cats of the World. Chicago: University of Chicago Press; 2002. Pp. 252-277.
81. Lima ES, DeMatteo KE, Jorge RSP, Jorge MLSP, Dalponte JC, Lima HS, et al. First telemetry study of *Speothos venaticus*: providing information on home range, activity, and habitat selection. Wildl Res. 2012;39(6):512-519.
82. Oliveira TG, Tortato MA, Silveira L, Kasper CB, Mazim FD, Lucherini M, et al. 2010. Chapter 27: Ocelot ecology and its effect on the small-felid guild in the lowland Neotropics. In Macdonald D, Loveridge A, editors. Biology and Conservation of Wild Felids. Oxford: Oxford University Press. Pp. 563-574.
83. Kasper CB, Schneider A, Oliveira TG. Home range and density of three sympatric felids in the Southern Atlantic forest, Brazil. Braz J. Biol. 2016;76:228-232. https://doi.org/10.1590/1519- 6989.19414.
84. Fielding AH, Bell JF. A review of methods for the assessment of prediction errors in conservation presence/absence models. Environ Conserv J. 1997;24:38-49.
85. Skov F, Borchsenius F. Predicting plant species distribution patterns using simple climatic parameters: a case study of Ecuadorian palms. Ecography. 1997;20:347-355.
86. Swets KA. Measuring the accuracy of diagnostic systems. Science. 1988;240:1285-1293.
87. Goulart FVB, Cáceres NC, Graipel ME, Tortato MA, Ghizoni Jr IR, Gustavo L, et al. Habitat selection by large mammals in a southern Brazilian Atlantic Forest. Mamm Biol. 2009;74:182-190.
88. Di Bitetti MS, De Angelo CD, Di Blanco YE, Paviolo A. Niche partitioning and species coexistence in a Neotropical felid assemblage. Acta Oecol. (Montrouge) 2010;36:403-412.
89. DeMatteo KE, Michalski F, Leite-Pitman MRP. *Speothos venaticus*. In: IUCN 2011. IUCN Red List of Threated Species. Version 2011.2. [<ww](http://www/)w[.](http://www/) Iucnredlist.org.
90. Cruz P, De Angelo C, Pardo JM, Iezzi ME, Varela D, Di Bitetti MS, et al. Cats under cover: habitat models indicate a high dependency on woodlands by Atlantic Forest felids. Biotopica 2019;51:266- 278.
91. Dellinger JA, Proctor C, Steury TD, Kelly MJ, Vaughan MR. Habitat selection of a large carnivore, the red world, in a human-altered landscape. Biol Conserv. 2013;157:324-330.
92. Gilbert-Norton L, Wilson R, Stevens JR, Beard KH. A meta-analytic review of corridor effectiveness.

Cons Biol. 2010;24(3):660-668.

93. Moilanen A, Arponen A. Setting conservation targets under budgetary constraints. Biol Conserv.

2010;144:650-653.

94. Jackson ST, Sax DF. Balancing biodiversity in a changing environment: extinction debt, immigration credit and species turnover. TREE 2009;25(3):153-160.
95. Wu Z, Pagell M. Balancing priorities: decision-making in sustainable supply chain management. J. Oper Manag. 2011;29:577-590.
96. Chen Y, Wang K, lin Y, Shi W, Song Y, He X. Balancing green and grain trade. Nat Geosci.

2015;8:739-741.

97. Gavin MC, McCarter J, Mead A, Berkes F, Stepp, JR, Peterson D, et al. Defining biocultural approaches to conservation. TREE 2015;30(3):140-145.
98. Winemiller KO, McIntyre PB, Castello L, Fluet-Chouinard, Giarrizzo T, Nam S, et al. Balancing hydropower and biodiversity in the Amazon, Congo, and Mekong. Science 2016;351(6269):128- 129.
99. Treves A, Karanth KU. Human-carnivore conflict and perspectives on carnivore management worldwide. Cons Biol. 2003;17(6):1491-1499.
100. Pettigrew M, Xie Y, Kang A, Roa M, Goodrich J, Liu T, et al. Human-carnivore conflict in China: a review of current approaches with recommendations for improved management. Integr Zool. 2012;7:210-226.
101. Rust NA, Marker LL. Cost of carnivore coexistence on communal and resettled land in Namibia.

Environ Conserv. 2013;41(1):45-53.

102. Young JK, Ma Z, Laudati A, Berger J. Human-carnivore interactions: lessons learned from communities in the American west. Hum Dimens Wildl. 2015;20(4):349-366. Doi: 10.1080/10871209.2015.1016388
103. Young JC, Marzano M, White RM, McCracken DI, Redpath, Carss DN, et al. The emergence of biodiversity conflicts from biodiversity impacts: characteristics and management strategies. Biodivers Conserv. 2010;19:3973-3990.
104. Veíssimo D. Influencing human behaviour: an underutilized tool for biodiversity management.

Conserv Evid. 2013;29-31.

105. Dilkina B, Houtman R, Gomes CP, Montgomery CA, McKelvey KS, Kendall K, et al. Trade-offs and efficiencies in optimal budget-constrained multispecies corridor networks. Cons Biol. 2017;31(1):192-202.
106. Di Minin E, Slotow, Hunter LTB, Pouzols FM, Toivonen T, Verburg PH, et al. Global priorities for national carnivore conservation under land use change. Sci Rep. 2016;6:23814. DOI: 10.1038/srep23814.
107. Peterson DL, Miller CL, Joyce LA, Furniss MJ, Halofsky JE, Neilson RP, et al. Responding to climate change in national forests: a guidebook to developing adaptation options. Gen. Tech. Rep. PNW-GTR-855. Portland OR: U.S. Department of Agriculture Forest Service, Pacific Northwest Research Station. 109 p.
108. Schwartz MW. Using niche models with climate projections to inform conservation management decisions. Biol Conserv. 2012;155:149-156.
109. Faleiro FV, Machado RB, Loyola RD. Defining spatial conservation priorities in the face of land- use and climate change. Biol Conserv. 2013;158:248-257.
110. Fick SE, Higmans. WorldClim 2: new 1-km spatial resolution climate surfaces for global land areas. Int J Climatol. 2017;37:4302-4315.

### Supporting Information

**S1 Appendix. Details of the 1085 scat swabs with confirmed species identity.** For the 1034 felid samples and 51 bush dog samples, the location and zone (North-Central) are summed by species. For protected areas, the total area is reported in parentheses.

| **Location** | **Zone** | **Jaguar** | **Puma** | **Ocelot** | **Southern tiger cat** | **Bush dog** |
| --- | --- | --- | --- | --- | --- | --- |
| Ejército Argentino (6,951 ha) | N | 2 | 2 | --- | 6 | 2 |
| Parque Nacional Iguazú (54,380 ha) | N | 24 | 13 | 27 | --- | --- |
| Parque Provincial (P.P.) Guardaparque H. Foerster (4,309 ha) | N | --- | --- | 6 | 6 | 1 |
| P.P. Puerto Península (6,900 ha) | N | 9 | 4 | 3 | 52 | 5 |
| P.P. Urugua-í (84,000 ha) | N | 13 | 9 | 17 | 37 | --- |
| Refugio Privado Aguaraí-mi (3,050 ha) | N | --- | 3 | --- | 22 | --- |
| Reserva de Vida Silvestre Urugua-í (3,243 ha) | N | --- | --- | 1 | 1 | --- |
| Reserva Nacional Iguazú (12,620 ha) | N | --- | --- | --- | 3 | --- |
| Reserva Natural Privada Yate-í (15 ha) | N | --- | --- | --- | 7 | --- |
| Reserva Privada Karadya (90 ha) | N | --- | --- | --- | 4 | --- |
| Reserva San Jorge (21,163 ha) | N | 5 | 3 | 5 | 22 | 1 |
| Reserva Yacutinga (539 ha) | N | --- | --- | 11 | --- | --- |
| P.P. Araucaria (92 ha) | C | --- | --- | --- | 1 | --- |
| P.P. Cruce Caballero (522 ha) | C | --- | 2 | 2 | 2 | --- |
| Reserva Privada Itaovy (80 ha) & Reserva Privada Yacutoro (200 ha) | C | --- | --- | --- | 3 | --- |
| Valle del Arroyo Alegría (8,000 ha) | C | --- | 2 | 2 | 39 | 1 |
| P.P. Cruce Caballero (522 ha) & Valle del Arroyo Alegría (8,000 ha) | C | --- | --- | --- | 2 | --- |
| P.P. Esmeralda (31,569 ha) & Reserva de Biósfera Yabotí (236,313 ha) | C | 3 | 19 | 28 | 56 | 5 |
| P.P. Piñalito (3,796 ha) | C | --- | --- | --- | 9 | 3 |
| Reserva Privada Yaguaroundí (400 ha) | C | --- | --- | --- | 5 | --- |
| outside protected areas | N | 17 | 14 | 37 | 197 | 20 |
| outside protected areas | C | 3 | 28 | 6 | 240 | 13 |
|  |  | **76** | **99** | **145** | **714** | **51** |

Figure 1a	[Click here to access/download;Figure;Figure 1a - GEF2015.jpg](https://www.editorialmanager.com/pone/download.aspx?id=31724592&guid=309c138d-1915-41fd-87fb-c94d96481dd9&scheme=1)

Figure 1b	[Click here to access/download;Figure;Figure 1b - MABF2018.jpg](https://www.editorialmanager.com/pone/download.aspx?id=31724593&guid=8c470640-07c5-44a9-a1f6-db2be99ca7f2&scheme=1)

Figure 2a	[Click here to access/download;Figure;Figure 2a - GEF2015 - SDM and PSR.jpg](https://www.editorialmanager.com/pone/download.aspx?id=31724596&guid=5d03f9af-0852-4949-a668-62f6839990d6&scheme=1)

Figure 2b	[Click here to access/download;Figure;Figure 2b - MABF2018 - SDM and PSR.jpg](https://www.editorialmanager.com/pone/download.aspx?id=31724614&guid=143502eb-d6a3-43ff-9d0a-e427dc0df782&scheme=1)

Figure 3a	[Click here to access/download;Figure;Figure 3a - GEF2015 - PSR - MOH.jpg](https://www.editorialmanager.com/pone/download.aspx?id=31724600&guid=aea61f81-8f84-46f3-9ba0-61e886ba4413&scheme=1)

Figure 3b	[Click here to access/download;Figure;Figure 3b - MABF2018 - PSR - MOH.jpg](https://www.editorialmanager.com/pone/download.aspx?id=31724602&guid=704e09c8-ff41-4c44-82b1-bfeba8b50b84&scheme=1)

Figure 4a	[Click here to access/download;Figure;Figure 4a - GEF2015 - PSR MOH1 - 2017 Connector.jpg](https://www.editorialmanager.com/pone/download.aspx?id=31724604&guid=67dbdffd-be47-4873-9a41-e0a0446b7c50&scheme=1)

Figure 4b	[Click here to access/download;Figure;Figure 4b - MABF2018 - PSR MOH - 2017 Connector.jpg](https://www.editorialmanager.com/pone/download.aspx?id=31724605&guid=2555d153-c7a1-4499-a390-13a6af70ae6f&scheme=1)

S1 - Appendix

[Click here to access/download](https://www.editorialmanager.com/pone/download.aspx?id=31724606&guid=acca1906-103b-4df6-9636-1af05db4e643&scheme=1)

[**Supporting Information** DeMatteo et al - S1 - Appendix.docx](https://www.editorialmanager.com/pone/download.aspx?id=31724606&guid=acca1906-103b-4df6-9636-1af05db4e643&scheme=1)
