# Supplementary material for: A multispecies corridor in a fragmented landscape: Evaluating effectiveness and identifying high-priority target areas
Source: PLoS One. 2023 Apr 13;18(4):e0283258. doi: 10.1371/journal.pone.0283258 (PMC10101518; doi:10.1371/journal.pone.0283258)
Supplement: Attachment — Submitted filename: Response to Reviewers.docx [file pone.0283258.s003.docx]

**Reviewer comments:**

All suggested word changes in the document were made, with a few exceptions. A general note: in the references at the end of the manuscript and in the appendix, there were multiple notes for formatting in Italian; however, no actual changes to the text were noted, so no changes were made in this section. The exceptions were:

1) Line 85: The use of “its” is appropriate in this case, as it indicated possessive, with Misiones being the region’s National Capital of Biodiversity. “It’s” would be incorrect, as an abbreviation of “it is” is not appropriate here.
2) Line 219: The use of “was” versus “were” is correct in this instance, as it is referring to the ‘neighborhood scale’, which is singular.
3) Line 240: The use of “was” versus “were” is correct in this instance, as it is referring to the ‘efficacy’, which is singular.
4) Line 267: The use of “fit” versus “fits” is appropriate and follows the rest of the paragraph where “fit” is considered appropriate.
5) Line 303: The text was not changed to “suitability levels reported”, as this would remove consistency for future sections in the Discussion (page 28 and page 30) where the results are discussed relative to “levels of suitability”. The change is a wording preference versus a change of meaning.

**Editor comments:**

1) Overlapping text: Per an email on 29 November 2022, Publication Assistant Alexis Miller noted that a review of the text found the overlap of text to be within the methods section. Therefore, the text of manuscript is acceptable under PLOS policy (Case 07798633).
2) An ethics statement was added, noting no verbal or written permission is required by the Saint Louis Zoo’s IACUC committee.
3) Question about copyrighted map images: Per an email on 7 December 2022, Publication Assistant Alexis Miller noted that a review of the journal policy did not find any potential copyright concerns regarding the figures in question. Therefore, the figures in the manuscript are acceptable under PLOS policy (Case 07798633).
4) Review of reference list: A review was made and no citations have been retracted.
